# Key challenges in prehospital and emergency care in Indonesia and Malaysia: a survey of frontline clinicians

**DOI:** 10.1186/s13104-024-06916-3

**Published:** 2024-10-03

**Authors:** Akio Tokita, Hanako Nunokawa, Keibun Liu, Yuta Iwamoto, Tomohiro Sonoo, Konan Hara, Mikio Nakajima, Kiyomitsu Fukaguchi, Takanori Takeda, Amirudin Sanip, Dafsah A. Juzar, a/l Harvendhar Singh Gurjeet Singh, Lukito Condro, Monalisa Tobing, Muhammad Abdus-Syakur bin Abu Hasan, Nik Hisamuddin Nik Abdul Rahman, Orizanov Mahisa, Ramdinal Aviesena Zairinal, Mohd Khairulizwan bin Ramli, Mohd Afiq Mohd Nor, Tadahiro Goto, Mohd Idzwan bin Zakaria

**Affiliations:** 1 School of Medicine, Faculty of Medicine, Gunma University, Gunma, Japan; 2 School of Medicine, Tokyo Medical and Dental University, Tokyo, Japan; 3 TXP Medical Co. Ltd., 7-3-1 Hongo, Bunkyo-Ku, Tokyo 113-0033 Japan; 4 Department of Economics, University of Arizona, Tucson, AZ USA; 5 Emergency Life-Saving Technique Academy of Tokyo, Foundation for Ambulance Service Development, Tokyo, Japan; 6 Department of Emergency Medicine, Shonan Kamakura General Hospital, Kanagawa, Japan; 7 Department of Emergency Medicine, Universiti Kebangsaan Malaysia, Kuala Lumpur, Malaysia; 8 Department of Cardiology and Vascular Medicine, Faculty of Medicine, Universitas Indonesia - National Cardiovascular Center Harapan Kita, Jakarta, Indonesia; 9 Hospital Selayang, Batu Caves, Malaysia; 10 RSUD Kanjuruhan Kabupaten Malang, Kepanjen, Indonesia; 11 Emergency Department, Hermina Depok Hospital, Depok, Indonesia; 12 Kulliyyah of Medicine, International Islamic University Malaysia, Kuantan, Malaysia; 13 Health Campus, Hospital Universiti Sains Malaysia, Kubang Kerian, Kota Bharu Kelantan, Malaysia; 14 Muhammadiyah Hospital Lamongan, Lamongan, Indonesia; 15 Head of Emergency Unit, Universitas Indonesia Hospital, Depok, Indonesia; 16 Department of Neurology, Faculty of Medicine, Universitas Indonesia, Jakarta, Indonesia; 17 Hospital Tengku Ampuan Afzan, Kuantan, Malaysia; 18 Department of Emergency Medicine, University Malaya Medical Center, Kuala Lumpur, Malaysia; 19 Academic Unit Trauma and Emergency, Faculty of Medicine, University of Malaya, Kuala Lumpur, Malaysia

**Keywords:** Ambulance, Emergency room, Indonesia, Malaysia, quality improvement

## Abstract

Rapid economic growth in Indonesia and Malaysia has widened the gap in emergency care supply and demand, intensifying challenges. Our study, from August to November 2022, assesses current diverse challenges in both countries' emergency care systems from frontline staff perspectives. The online survey involved emergency department (ED) personnel from 11 hospitals in Indonesia and Malaysia, drawing from an existing network. The survey collected data on respondents’ characteristics, factors affecting prehospital and ED care quality, missing clinical information, and factors influencing patients’ ED stay duration. With 83 respondents from Indonesia and 109 from Malaysia, the study identified common challenges. In both countries, inadequate clinical information from ambulances posed a primary challenge in prehospital care quality, while crowdedness during night shifts affected ED care quality. Frequent gaps in essential clinical information, such as family and medication history, were observed. Prolonged ED stays were associated with diagnostic studies and their turnaround time. This study offers insights into shared challenges in Indonesia and Malaysia's emergency care systems. Our findings stress recognizing common and country-specific challenges for enhanced emergency care quality in Southeast Asia, supporting tailored interventions.

**Supplementary Information:**

The online version contains supplementary material available at 10.1186/s13104-024-06916-3.

## Introduction

Recent economic growth in Southeast Asia has widened the gap between the supply and demand for emergency care, potentially leading to poorer health outcomes [1]. In Indonesia and Malaysia, traffic accident mortality rates per 100,000 population are significantly higher (12.2 and 23.6, respectively) compared to developed Asia–Pacific countries like Singapore (2.8), Australia (5.6), and Japan (4.1) [2]. The growing demand for emergency care highlights the need for improvement in the emergency care systems.

Studies have shown the current inadequacy of emergency care in Indonesia and Malaysia. For instance, in Indonesia, 62.8% of myocardial infarction patients failed to receive timely reperfusion therapy because they arrived at the emergency department (ED) over 12 h after symptom onset [3]. In Malaysia, the average “door-to-needle” time for acute myocardial infarction (time from ED arrival to initiation of thrombolytic therapy) averaged 105 min, exceeding the time of less than 30 min to improve outcomes [4].

In addition to system-level issues, both countries face its own challenges in emergency care education and the number of specialists. Emergency medicine was officially recognized as a specialty in Indonesia in 2017, with a severe shortage of trained emergency physicians affecting care. Most emergency care is provided by general practitioners without specialized training [5]. On the other hand, Malaysia has long had a well-developed educational system for emergency medicine, with a system of emergency medical specialists and a system of paramedics and nurses (as detailed in Appendix 2 of the Supplementary Material). However, Malaysia still has only 7.4 certified emergency physicians per million population, less than half the number in Singapore [1]. Additionally, in Malaysia, there are currently no standardized educational courses for prehospital care providers [6]. To make matters worse, both countries face barriers such as the lack of a national consensus on ambulance triage protocols, insufficient ambulance services, and low priority given to ambulances in traffic congestion, which impede the development of effective emergency care [7, 8].

In this context, understanding these prioritized issues is vital for implementing effective interventions and achieving systemic improvements. Our study aimed to elucidate the prevailing issues within their emergency care systems of Indonesia and Malaysia, as identified by frontline ED staff, informing initiatives of developing targeted interventions to enhance the efficiency and effectiveness of emergency care systems.

## Materials and methods

### Design

This cross-sectional study was conducted online using Google Forms (Alphabet Inc., California, USA) from August to november 2022, involving five hospitals in indonesia and six in malaysia. These hospitals were selected through existing research networks. The names and locations of the hospitals are presented in supplemental Fig. 1. Ethical approval was waived by the central ethics committee of txp medical. co. ltd, while local ethical approval were obtained at each site. All experimental protocols were approved from the respective local ethics committee. The study adheres to the consensus-based checklist for reporting of survey studies (cross) guidelines for survey research. [9]

**Fig. 1:**
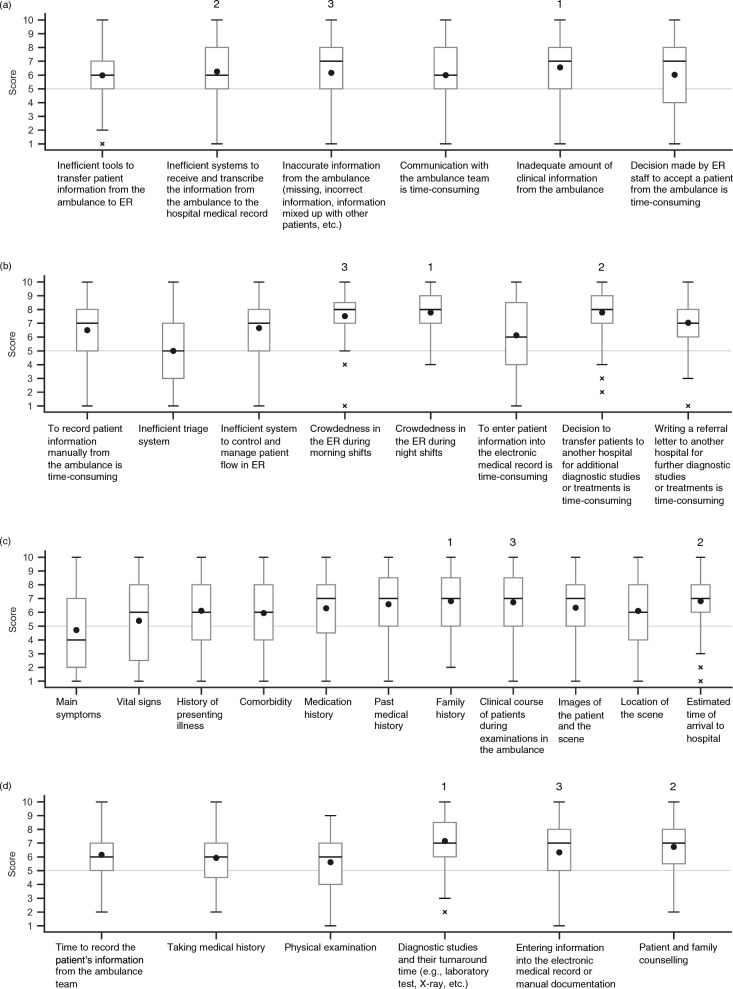
Box plots of survey results for emergency care issues in Indonesia. The lines inside each boxplot represent the median value and the circles inside each boxplot represent the mean value. The top three highest mean values in each boxplot are labeled 1, 2, and 3 above the corresponding boxes. **a** Issues identified in the quality of prehospital care. Scale: 1 = Strongly Disagree, 10 = Strongly Agree. **b** Issues identified in the quality of emergency care in the ED. Scale: for the first question, 1 = Strongly Disagree, 10 = Strongly Agree; for the second question, 1 = Least crowded, 10 = Most crowded; for the last two questions, 1 = Very easy, 10 = Time-consuming. **c** Missing clinical information from prehospital care to the ED. Scale: 1 = Strongly Disagree, 10 = Strongly Agree. **d** Issues identified in the length of patient stay at the ED. Scale: 1 = Very short, 10 = Very long. ED, emergency department

The url of the online questionnaire was distributed to all ed staff, including doctors, nurses, and medical workers, by each site’s chief investigator. Throughout the study period, staff were prohibited from discussing the survey content. The survey’s purpose and respondent rights were detailed on the first page of the questionnaires (see supplementary material). Invited ED staff who agreed with the survey policy and provided informed consent were directed to the questionnaire webpage. Participation was anonymous, voluntary, and uncompensated.

### Survey settings

The survey collected diverse perspectives from ED staff in each country. A total of 11 hospitals, five from Indonesia and six from Malaysia, participated in the study. Details on the number of employees, hospital type, capacities, and patient volumes in Indonesia and Malaysia are listed in Supplemental Table 1.

**Table 1:** Characteristics of respondents

Characteristic	Indonesia (n = 83)	Malaysia (n = 109)
Male	22 (27)	56 (51)
Age (year)		
20–29	49 (59)	38 (35)
30–39	24 (29)	61 (56)
40–49	10 (12)	7 (6)
50–59	0 (0)	3 (3)
Occupation		
Assistant medical officer	0 (0)	41 (38)
Emergency physician (certified as a specialist in emergency medicine)	18 (22)	2 (2)
Emergency physician (non-certified)	10 (12)	0 (0)
Medical officer	1 (1)	47 (43)
Nurse	48 (58)	19 (17)
Pharmacist or health worker in pharmacy	4 (5)	0 (0)
Other	2 (2)	0 (0)
Experience in the current position (year)		
< 1	2 (2)	6 (6)
1–4	45 (54)	36 (33)
5–8	13 (16)	32 (29)
> 8	23 (28)	35 (32)
Highest academic qualification		
Diploma	26 (31)	57 (53)
Master’s degree	2 (2)	5 (5)
Undergraduate degree	42 (51)	47 (43)
Others	13 (16)	0 (0)

“Emergency physician (certified as a specialist in emergency medicine)" means a medical specialist who has successfully completed a predetermined course of study in emergency medicine; "Emergency physician (non-certified)" means a non-specialist physician; "medical officer" means a resident; "assistant medical officer" means a paramedic

### Contents of the online survey and measurements

The survey consists of two main sections: (1) Respondent Demographic Characteristics, which collected participant data, and (2) Survey on the Emergency Department, which aimed to investigate the issues associated with ED management and identify issues recognized by the participants. The questions were initially produced in English with the help of a native English speaker, then translated into the lingua franca in each country: Bahasa Indonesia for Indonesia and Bahasa Malaysia for Malaysia. We performed translation and back translation, and the consistency was confirmed by two emergency physicians (TG and LK). The questionnaire was formulated through more than 10 meetings, primarily based on the clinical experiences and expertise of our research team, aiming to encompass a wide array of issues commonly encountered in emergency care settings.

We collected the following information: (1) respondent characteristics (five questions), (2) issues identified in the quality of prehospital care (six questions), (3) issues identified in the quality of emergency care in the ED (eight questions), (4) missing clinical information from prehospital care or ambulance team to the ED (11 questions), and (5) issues identified in the length of patient stay at the ED (six questions). The questionnaire consisted of 47 questions, estimated to take approximately 10 min (see Supplementary material).

To prevent duplicated responses, an initial question asked if respondents had previously participated, with a prompt to continue or exit based on their answer. Furthermore, to ensure unique identification and prevent multiple entries, respondents were requested to provide the first five characters of their email addresses. These measures aimed to maintain the integrity and validity of the survey data by minimizing duplicate responses. All questions were configured as mandatory in the Google Form used for data collection, ensuring that respondents completed each item before proceeding. This methodology resulted in no missing responses.

### Statistical analysis

The characteristics were reported as medians and interquartile ranges (IQRs) for continuous variables and as numbers and percentages (%) for categorical variables, respectively. Issues identified in prehospital and emergency care, which were collected using a 10-point Likert scale, were analyzed as medians with IQRs and means, along with standard deviations [10, 11]. The Likert scale is simple to use and quantifies subjective opinions, allowing for nuanced responses and statistical analysis. Its consistency and flexibility make it a versatile tool in research. The mean values were used to identify the top three issues identified in prehospital and emergency care, because the respective median values of four or more items tied for the top ranking, making it challenging to discern the top three factors. Results are presented first for Indonesia, then Malaysia, ending with the common top issues in both countries. All analyses were performed using R version 4.2.1 (R Foundation for Statistical Computing, Vienna, Austria).

## Results

### Indonesia

#### Characteristics of respondents in Indonesia

In Indonesia, we obtained responses from 83 participants from five hospitals. As shown in Table 1, 27% were male, mostly aged 20–29 (59%), then 30–39 (29%). Nurses were 58% of respondents, with emergency physicians at 22%. Approximately half had 1–4 years of experience in the current position (54%). The highest academic qualification was an undergraduate degree (51%), followed by a diploma (31%).

### Issues identified in the quality of prehospital care in Indonesia

All items had a median score of 6 (of 10) or higher (higher score indicates more problematic issue) (Fig. 1a), indicating that the participants felt that there were several issues with the quality of prehospital care. The most important issue was “inadequate amount of clinical information from the ambulance (mean 6.57 ± 2.24; Table 2)”.

**Table 2:** Top 3 issues identified in with the delivery of emergency care

	Indonesia	Malaysia
Issues identified in with the quality of prehospital care		
Top 1	Inadequate amount of clinical information from the ambulance (median, 7 [IQR, 5–8])	Inadequate amount of clinical information from the ambulance (median, 5 [IQR, 4–7])
Top 2	Inefficient systems to receive and transcribe the information from the ambulance to the hospital medical record (median, 6 [IQR, 5–8])	Inefficient tools to transfer patient information from the ambulance to ER (median, 5 [IQR, 4–6])
Top 3	Inaccurate information from the ambulance (missing, incorrect information, information mixed up with other patients, etc.) (median, 7 [IQR, 5–8])	Inefficient systems to receive and transcribe the information from the ambulance to the hospital medical record (median, 5 [IQR, 4–7])
Issues identified in the quality of emergency care in the ED		
Top 1	Crowdedness in the ER during night shifts (median, 8 [IQR, 7–9])	Crowdedness in the ER during night shifts (median, 8 [IQR, 6–9])
Top 2	Decision to transfer patients to another hospital for additional diagnostic studies or treatments is time-consuming (median, 8 [IQR, 7–9])	Decision to transfer patients to another hospital for additional diagnostic studies or treatments is time-consuming (median, 8 [IQR, 6–9])
Top 3	Crowdedness in the ER during morning shifts (median, 8 [IQR, 7–9])	Writing a referral letter to another hospital for further diagnostic studies or treatments is time-consuming (median, 7 [IQR, 5–8])
Missing clinical information from prehospital care to the ED		
Top 1	Family history (median, 7 [IQR, 5–9])	Family history (median, 7 [IQR, 5–9])
Top 2	Estimated time of arrival to hospital (median, 7 [IQR, 6–8])	Medication history (median, 7 [IQR, 4–8])
Top 3	Clinical course of patients during examinations in the ambulance (median, 7 [IQR, 5–9])	Images of the patient and the scene (median, 6 [IQR, 3–9])
Issues identified in the length of patient stay at the ED		
Top 1	Diagnostic studies and their turnaround time (e.g., laboratory test, X-ray, etc.) (median, 7 [IQR, 6–9])	Diagnostic studies and their turnaround time (e.g., laboratory test, X-ray, etc.) (median, 8 [IQR, 7–9])
Top 2	Patient and family counselling (median, 7 [IQR, 6–8])	Patient and family counselling (median, 6 [IQR, 5–7])
Top 3	Entering information into the electronic medical record or manual documentation (median, 7 [IQR, 5–8])	Entering information into the electronic medical record or manual documentation (median, 5 [IQR, 4–7])

IQR, interquartile range; ER, emergency room; ED, emergency department

### Issues identified in the quality of emergency care in the ED in Indonesia

All items had a median score of 6 (of 10) or higher (Fig. 1b), indicating that the participants felt that there were several issues with the quality of patient care in the ED. The most important issues were “crowdedness in the emergency room (ER) during night shifts (7.78 ± 1.57; Table 2)” and “decision to transfer patients to another hospital for additional diagnostic studies or treatments is time-consuming (7.78 ± 1.79).” In contrast, about half of the participants believed the current triage system was sufficient (median 5 for “inefficient triage system”).

### Failure to transfer clinical information from prehospital care to the ED in Indonesia

Most clinical information from the prehospital care or the ambulance team to the ED (e.g., family history, estimated time of arrival to hospital, clinical progress of patient during the ambulance examinations) had a median score of 6 or higher, which indicates that relevant information was not adequately transferred to the ED, with the exception of information on main symptoms, which was relatively well transferred (median 4; Fig. 1c and Table 2). The clinical information with the highest score (least likely to be transferred from prehospital care to the ED) was family history (mean 6.81 ± 2.21; Table 2) and estimated time of arrival to hospital (6.81 ± 2.24).

### Issues identified in the length of patient stay at the ED in Indonesia

All factors had a score of 6 or higher (Fig. 1d). Among them, “diagnostic studies and their turnaround time (e.g., laboratory test, X-ray, etc.) (mean 7.16 ± 1.98; Table 2),” “patient and family counseling (6.72 ± 1.97),” and “entering information into the electronic medical record or manual documentation (6.33 ± 2.13)” had the highest scores in this order (Table 2). These findings suggest that the participants had concerns about various aspects of the ED processes that were causing extended patient stays.

## Malaysia

### Characteristics of respondents in Malaysia

In Malaysia, 109 respondents from six hospitals participated (Table 1). A majority of the respondents were male (51%), were between 30 and 39 (56%), and were Medical Officer (non-specialist doctor) or Assistant Medical Officer (medical assistant) (82%). Experience was equally distributed into 1–4, 5–8, and more than 8 years. The highest academic qualification was a diploma (53%), followed by an undergraduate degree (43%).

### Issues identified in the quality of prehospital care in Malaysia

**Fig. 2:**
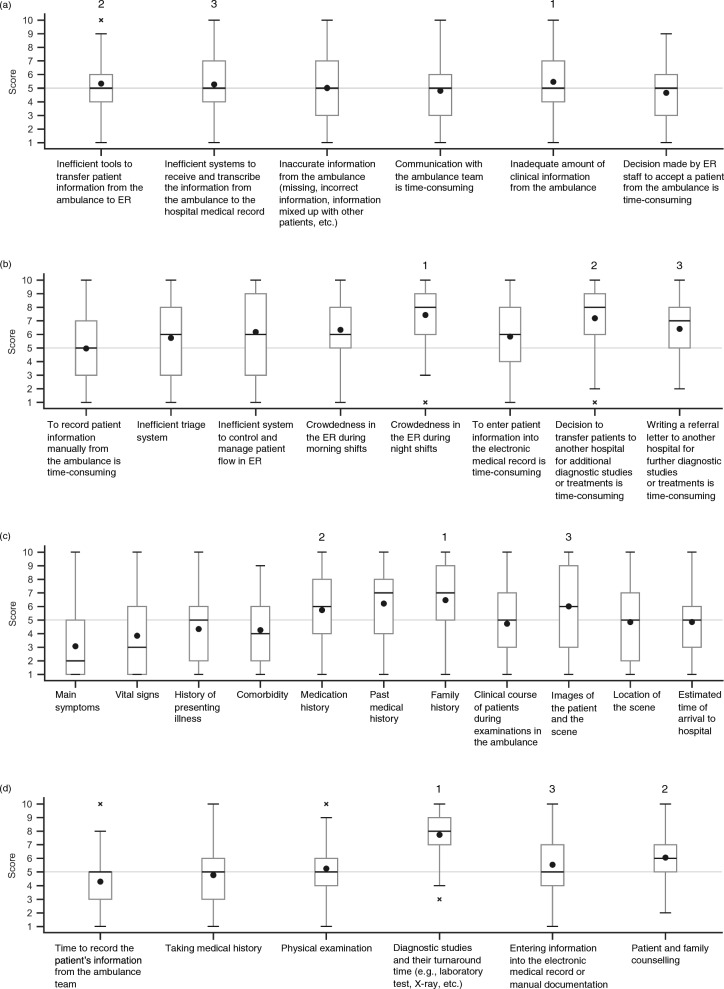
Box plots of survey results for emergency care issues in Malaysia. The lines inside each boxplot represent the median value and the circles inside each boxplot represent the mean value. The top three highest mean values in each boxplot are labeled 1, 2, and 3 above the corresponding boxes. **a** Issues identified in the quality of prehospital care. Scale: 1 = Strongly Disagree, 10 = Strongly Agree. **b** Issues identified in the quality of emergency care in the ED. Scale: for the first question, 1 = Strongly Disagree, 10 = Strongly Agree; for the second question, 1 = Least crowded, 10 = Most crowded; for the last two questions, 1 = Very easy, 10 = Time-consuming. **c** Missing clinical information from prehospital care to the ED. Scale: 1 = Strongly Disagree, 10 = Strongly Agree. **d** Issues identified in the length of patient stay at the ED. Scale: 1 = Very short, 10 = Very long. ED, emergency department

All items had a median score of 5 (Fig. 2a). The most important factor was “inadequate amount of clinical information from the ambulance (mean 5.46 ± 2.51; Table 2).” Overall, the participants had concerns about various aspects of the quality of patient care in the prehospital setting.

### Issues identified in the quality of emergency care in the ED in Malaysia

Most items had a median score of 6 of 10 or higher, reflecting a general perception of these factors as problematic issues (Fig. 2b). The most important issues were “crowdedness in the ER during night shifts (mean 7.43 ± 2.04; Table 2)” and “decision to refer patients to another hospital for additional diagnostic studies or treatments is time-consuming (7.19 ± 2.36).” In contrast, about half of the participants felt that the current triage system was sufficient, as indicated by a median score of 5 for “to record patient information manually from the ambulance is time-consuming.”

### Failure to transfer clinical information from prehospital care to the ED in Malaysia

Most clinical information from the prehospital care or the ambulance team to the ED (e.g., family history, estimated time of arrival to hospital, clinical course of patients during examinations in the ambulance) had a median score of 6 or higher, which indicates that relevant information was not adequately transferred to the ED, with the exception of information on main symptoms, which was relatively well transferred (Fig. 2c and Table 2). The clinical information with the highest score (least likely to be transferred from prehospital care to the ED) was family history (mean 6.47 ± 2.81).

### Issues identified in the length of patient stay at the ED in Malaysia

All factors had a median score of 5 or higher (Fig. 2d); participants thought there were several factors contributing to prolonged stay in the ED. The highest factors were “diagnostic studies and their turnaround time (mean 7.73 ± 1.84; Table 2),” “patient and family counselling (6.06 ± 1.76),” and “entering information into the electronic medical record or manual documentation (5.53 ± 2.09).”

## Factors commonly recognized in both Indonesia and Malaysia

Among the top three factors of each section (issues identified in the quality of prehospital care, issues identified in the quality of emergency care in the ED, missing clinical information from prehospital care to the ED, and issues identified in with the length of patient stay at the ED), factors with the highest scores were the same between both countries: “inadequate amount of clinical information from the ambulance,” “crowdedness in the ER during night shifts,” “family history,” and “diagnostic studies and their turnaround time,” respectively.

## Occupational differences among respondents in Indonesia and Malaysia

In comparing the occupations of respondent participants from Indonesia and Malaysia, distinct differences were evident. In Malaysia, the survey revealed a significant representation of Assistant Medical Officers (38%), reflecting a more structured emergency medical systems with specialized roles. Conversely, Indonesia had no respondents in these categories. Instead, the majority of Indonesian respondents were nurses (58%). The reason for the absence of Assistant medical officers in Indonesia is that the role of paramedics is filled by nurses, resulting in an occupational imbalance. Additionally, Indonesia had respondents from other occupations (2%) and pharmacists or health workers in pharmacy (5%), categories that were not represented in Malaysia. These differences highlight the varied stages of emergency medical systems development and professionalization in the two countries.

## Discussions

We identified several issues in the current prehospital and emergency care in Indonesia and Malaysia that should be targeted for improvement to enhance the quality of care and the overall healthcare system. This survey represents the initial attempt to uncover issues affecting the quality of emergency care, as recognized by frontline ED staff.

Despite the differences in the prevalence of emergency systems and the course of specialty training between the two countries (as detailed in Appendix 1 and 2 of the Supplementary material), we found common issues. In fact, the distribution of prehospital care quality scores, including the median, was slightly skewed toward the smaller end of the scale in Malaysia. This could reflect the greater recognition and prevalence of prehospital care in Malaysia. However, in both countries, the important factors affecting the quality of patient care in the prehospital setting were “inadequate amount of clinical information from the ambulance” and “inefficient systems to receive and transcribe the information from the ambulance to the hospital medical record.” Ambulance teams are tasked with a multitude of responsibilities, including patient care, gathering patient information, communicating with the ED, and monitoring the patient during transport [12]. The challenge lies in balancing the collection of clinical information with the time available for patient care [13]. In severe cases, the priority is to provide immediate care and treatment, which may limit the time available for additional information collection [13].

To address these issues, a novel and innovative approach for data collection and transfer could be a solution. For instance, using smartphone applications to transfer data from ambulance teams to hospitals could reduce communication time with the ED [14]. Additionally, the use of optical character recognition or voice recording to capture clinical information (e.g., vital signs) from ambulance teams or the automatic transfer of data from the vital sign monitors in the ambulance to the ED could reduce the risk of missing information [15]. The crowdedness in the ER during night shifts was rated as the highest issue affecting quality of patient care. Although optical character recognition and voice recording could streamline the process of inputting information into electrical medical records [14], it may not necessarily improve the turnaround time for diagnostic studies or the time required for patient and family counseling. Given that “diagnostic studies and their turnaround time (e.g., laboratory test, X-ray, etc.)” was recognized as a factor associated with the length of patient stay at the ED, more robust approaches that enable immediate data sharing across departments in the hospital might be beneficial in alleviating crowding. Importantly, despite differences in their training backgrounds, medical cultures, and practice environments, ED staff demonstrated similar ratings of issues affecting patient length of stay in the ED.

Interestingly, this study demonstrated that issues prioritized by staff were consistent across both countries. The best approach may be to first explore the solution that can address these issues. In addition, these factors may be prevalent in other developing countries, so solutions may be effective in these countries as well. In contrast, some factors varied across the two countries, suggesting that factors affecting the quality of prehospital and emergency care could vary widely across countries. This also highlights the importance of tailoring interventions based on the specific background and situation of each setting and that a one-size-fits-all solution is unlikely to be effective [16, 17].

## Limitations

This study has several limitations. First, the survey questionnaire was developed from the team's experiences without pre-testing, which might have led to misinterpretation that could affect response accuracy. Additionally, the lack of pre-testing of the questionnaire could have limited the depth of insight into issues such as overcrowding. Future studies should aim for more in-depth exploration of specific issues by conducting pilot tests to increase the value of the data collected and provide more actionable insights. Second, the survey reflects only the perceptions of hospital ED staff, possibly differing significantly from actual clinical practice. This discrepancy emphasized the need for further research to validate these findings against patient outcomes. Third, we could not ascertain the total survey distribution, preventing response rate calculation and potentially introducing non-responder and selection bias. For example, most respondents in Indonesia were nurses, while in Malaysia, they were primarily medical officers. No information was obtained on whether the respondents were general practitioners or not. Additionally, there were also potential biases due to the uneven distribution of emergency physicians between the two countries and the almost complete absence of medical officers in Indonesia. In both countries, the proportion of respondents from national vs. private hospitals was unknown, which could have biased responses regarding ED crowding. Finally, the number of participating hospitals and respondents was also limited, with hospitals selected from existing networks, not randomly, which could bias results. Consequently, caution is needed when extrapolating these findings to actual clinical outcomes and broader hospital landscape in these regions. Conducting large-scale surveys is quite challenging in developing countries due to the absence of established large-scale survey panels. Despite these difficulties, this study attempted to overcome these challenges and can still detect potential priority areas for intervention.

## Conclusions

This survey identified the challenges faced by frontline staff in providing quality emergency care in Indonesia and Malaysia. Our results identified top priority factors to be addressed in prehospital and emergency care, such as lack of information from ambulance services, overcrowding in the ED, and prolonged time of input into medical records. This study not only provides a snapshot of current challenges but also serves as a foundation for developing targeted interventions for the specific needs of Indonesia and Malaysia. By identifying the top priority factors, such as communication gaps (e.g., family history, medication history, and predicted time of hospital arrival) in prehospital care and night shift overcrowding, our research can lead to intervention trials that could significantly improve emergency care outcomes in these countries. Future research should focus on targeted interventions to address these challenges and evaluate their effectiveness in improving emergency care outcomes.

## Supplementary Information

Additional file1 (DOCX 420 KB)

## Data Availability

The datasets generated and/or analyzed during the current study are available from the corresponding author on reasonable request.
