# Supplementary material for: Key challenges in prehospital and emergency care in Indonesia and Malaysia: a survey of frontline clinicians
Source: BMC Res Notes. 2024 Oct 3;17:287. doi: 10.1186/s13104-024-06916-3 (PMC11448250; doi:10.1186/s13104-024-06916-3)
Supplement: Supplementary file 1 — Additional file1 (DOCX 420 KB) [file 13104_2024_6916_MOESM1_ESM.docx]

**Supplementary material**

**Supplemental Table 1. Characteristics of participating hospitals**

| **Characteristic** | **Indonesia**  **(n=5)** | **Malaysia**  **(n=6)** |
| --- | --- | --- |
| Total number of employees in the hospital | 778 [700, 1039] | 3750 [3550, 4025] |
| Level of healthcare |  |  |
| Secondary | 4 (80) | 0 (0) |
| Tertiary | 1 (20) | 6 (100) |
| Type of hospital |  |  |
| National hospital | 3 (60) | 5 (83) |
| Private hospital | 2 (40) | 1 (17) |
| Number of hospital beds | 125 [113, 138] | 1054 [969, 1127] |
| Number of beds in ED | 25 [21, 29] | 51 [34, 56] |
| Number of patients per month transferred to ED | 1825 [1336, 1922] | 6130 [6020, 6930] |
| Number of ED patients per month transferred by local ambulance from the scene | 163 [82, 420] | 934 [778, 1138] |
| Number of ED patients per month transferred by private car from the scene | 800 [530, 1150] | 5000 [5000, 5500] |
| Number of ED patients per month transferred from other medical facilities (patient transport between hospitals) | 152 [84, 270] | 125 [39, 300] |

Data in the table are presented as median [interquartile range] or number (percentage). The hospital information was surveyed in July 2022.

Abbreviations: ED, emergency department

**Supplemental Table 2. Top 3 issues identified in with the delivery of emergency care (means and standard deviations shown)**

|  | **Indonesia** | **Malaysia** |
| --- | --- | --- |
| **Issues identified in with the quality of prehospital care** | | |
| Top 1 | Inadequate amount of clinical information from the ambulance (6.57±2.24) | Inadequate amount of clinical information from the ambulance (5.46±2.51) |
| Top 2 | Inefficient systems to receive and transcribe the information from the ambulance to the hospital medical record (6.27±2.11) | Inefficient tools to transfer patient information from the ambulance to ER (5.33±2.27) |
| Top 3 | Inaccurate information from the ambulance (missing, incorrect information, information mixed up with other patients, etc.) (6.17±2.29) | Inefficient systems to receive and transcribe the information from the ambulance to the hospital medical record (5.28±2.19) |
| **Issues identified in** **the quality of emergency care in the ED** | | |
| Top 1 | Crowdedness in the ER during night shifts (7.78±1.57) | Crowdedness in the ER during night shifts (7.43±2.04) |
| Top 2 | Decision to transfer patients to another hospital for additional diagnostic studies or treatments is time-consuming (7.78±1.79) | Decision to transfer patients to another hospital for additional diagnostic studies or treatments is time-consuming (7.19±2.36) |
| Top 3 | Crowdedness in the ER during morning shifts (7.52±1.55) | Writing a referral letter to another hospital for further diagnostic studies or treatments is time-consuming (6.41±2.30) |
| **Missing clinical information from prehospital care** **to the ED** | | |
| Top 1 | Family history (6.81±2.21) | Family history (6.47±2.81) |
| Top 2 | Estimated time of arrival to hospital (6.81±2.24) | Medication history (6.22±2.71) |
| Top 3 | Clinical course of patients during examinations in the ambulance (6.72±2.26) | Images of the patient and the scene (6.01±3.06) |
| **Issues identified in the length of patient stay at the ED** | | |
| Top 1 | Diagnostic studies and their turnaround time (e.g., laboratory test, X-ray, etc.) (7.16±1.98) | Diagnostic studies and their turnaround time (e.g., laboratory test, X-ray, etc.) (7.73±1.84) |
| Top 2 | Patient and family counselling (6.72±1.97 [IQR, 6-8]) | Patient and family counselling (6.06±1.76) |
| Top 3 | Entering information into the electronic medical record or manual documentation (6.33±2.13) | Entering information into the electronic medical record or manual documentation (5.53±2.09) |

Data in the table are presented as mean ± SD

Abbreviations: SD, standard deviation; ER, emergency room; ED, emergency department

**Appendix 1. Differences between Indonesian and Malaysian emergency medical care systems**

The emergency medical systems in Malaysia and Indonesia differ significantly in ambulance use and emergency department (ED) visits due to infrastructure, public awareness, and healthcare policies.

**Malaysia:**

Malaysia has a well-developed ambulance service infrastructure. As of 2017, there were 2,039 ambulances, with 55.2% in hospitals and 44.8% in health clinics. Demand for ambulance services has been increasing, particularly due to rising road traffic accidents, with a reported rate of 17.4 per 1,000 population in 2019.[1] Ambulance utilization is higher in health clinics than in hospitals due to the referral system. Clinics reported a median of 31.9 trips and 58.6 hours of ambulance usage per month, while hospitals reported 16.3 trips and 39.3 hours.[2] Emergency department visits in Malaysia are influenced by the structured referral system from health clinics to hospitals. Clinics act as gatekeepers, and their ambulance services are heavily utilized for patient transfers to hospitals. The number of ED visits has been increasing significantly, leading to overcrowding issues. A substantial portion of these visits are for non-critical cases, with reports indicating that 55% to 62.1% of ED visits are inappropriate.[3,4]

**Indonesia:**

In Jakarta, approximately 2 million people are seen in the ED annually, 202 per 1,000 population.[5] Compared to Malaysia, ED consultations show a different pattern. Ambulance services in Indonesia are less utilized; in Jakarta, only 9.3% of patients use ambulances, primarily due to lack of awareness, high costs, and long response times (median: 24 minutes).[6] Only 10% of emergency transportation in tertiary hospitals involved ambulances, with most patients arriving via private cars or ride-sharing services.[7] Many visits are self-admitted due to underdeveloped pre-hospital services, leading to prolonged ED stays and higher in-hospital mortality.[8]

There are several types of ambulances in Indonesia, including those provided by the Public Safety Center (PSC 119), which was developed in 2016. PSC 119 ambulances are government-operated and staffed with medical teams that provide pre-hospital emergency medical services. This system works well in big cities like Jakarta, Yogyakarta, and Magelang, where patient medical records are provided to inform EDs. Additionally, there are community/NGO-based ambulances (e.g., Ambulance Muhammadiyah, Ambulance Nahdlatul Ulama), hospital-based ambulances, and even ambulances provided by politicians. Also, Indonesia has a referral patient application called Sistem Rujukan Terintegrasi (SISRUTE) that helps store and inform patients' conditions to referral hospitals. However, the overall utilization of emergency services is still developing, with many patients unaware of how to access ambulance services. The low prevalence of ambulance use indicates a need for improved public awareness and accessibility of emergency medical services.

**Summary:**

Malaysia has a more developed and utilized ambulance service system compared to Indonesia, where ambulance use is still low due to various challenges. Emergency department visits in Malaysia are better managed through a structured referral system, while in Indonesia, there is significant reliance on private transportation for emergency care. Both countries face high prevalence rates of ED visits, but Malaysia's EDs are overwhelmed by non-critical cases, while Indonesia's EDs struggle with prolonged length of stay and admitted cases due to inadequate pre-hospital services.

**Supplemental Table 3. Comparison in emergency medical care systems: Malaysia vs. Indonesia**

| Aspect | Malaysia | Indonesia |
| --- | --- | --- |
| Ambulance Utilization | Higher in clinics (31.9 trips/month) than hospitals (16.3 trips/month) [2]  Structured referral system from clinics to hospitals. Higher ambulance uses in clinics vs. hospitals [2] | Lower, with only 9.3% of patients using ambulances, with most patients using private vehicles [6] |
| Annual ED Visits | Increasing significantly, leading to overcrowding. 55% to 62.1% of visits are non-critical [4] | Approximately 2 million in Jakarta, 202 per 1,000 people [5] |
| ED Overcrowding | Significant overcrowding issues [3] | Prolonged ED length of stay and overcrowding [8] |

**[References]**

1. Front Public Health. 2023 Jan 6:10:959812. doi: 10.3389/fpubh.2022.959812. eCollection 2022.

2. PLoS One. 2022 Nov 4;17(11):e0276632. doi: 10.1371/journal.pone.0276632. eCollection 2022.

3. BMC Health Serv Res. 2022 May 13;22(1):639. doi: 10.1186/s12913-022-08061-8.

4. <https://www.medrxiv.org/content/10.1101/2024.01.18.24301470v1.full> doi: https://doi.org/10.1101/2024.01.18.24301470

5 Emerg Med Australas. 2020 Oct;32(5):830-839. doi: 10.1111/1742-6723.13570. Epub 2020 Jul 31.

6..<https://www.researchgate.net/publication/262357567_Frequency_of_admittance_and_probability_of_inpatient_treatment_Experience_of_Emergency_Department_Hospital_Universiti_Kebangsaan_Malaysia>

7. <https://journals.utm.my/index.php/jurnalteknologi/article/view/8297> doi: 10.11113/jt.v78.8297

8. Open Access Emerg Med. 2023 Sep 13:15:313-323. doi: 10.2147/OAEM.S415971. eCollection 2023.

**Appendix 2. Differences in training of emergency medicine specialists in Malaysia and Indonesia**

**Malaysia:**

Malaysia's postgraduate training for emergency medicine includes the Master of Emergency Medicine (MEmMed) program introduced in 2005, supported by the Ministry of Health, ensuring consistent training.[1] Multiple pathways like MEmMed and Fellowship of the Royal College of Emergency Medicine (FRCEM) exams offer flexibility. Government through scholarships and paid leave support has significantly increased the number of emergency physicians.[2]

**Indonesia:**

Indonesia's emergency medicine training is developing, with initiatives like a multi-country training hub in collaboration with the World Health Organization, focusing on comprehensive training to improve specialist prevalence and quality.[3,4]

**Summary:**

Malaysia has an established system for training emergency medicine specialists with substantial government support. Indonesia is building its infrastructure with collaborative efforts to enhance training quality and prevalence.

**Supplemental Table 4. Comparison in training of emergency medicine specialists: Malaysia vs. Indonesia**

| Aspect | Malaysia | Indonesia |
| --- | --- | --- |
| Established Programs | Well-established with structured postgraduate programs (e.g., MEmMed) | Indonesia's emergency medicine training is developing, with initiatives like a multi-country training hub in collaboration with the World Health Organization [3,4] |
| National Curriculum | Standardized by the National Postgraduate Medical Curriculum | No unified national curriculum, but collaborative efforts with WHO |
| Support and Growth | Significant support from the Ministry of Health, leading to increased specialists | Limited awareness and utilization, but strategic initiatives underway |
| Training Pathways | Multiple pathways (master’s degree, FRCEM) | Focus on collaborative training hubs and international partnerships |

**[References]**

1. https://medicine.um.edu.my/master-of-emergency-medicine

2. https://medicine.um.edu.my/pdf/postgraduate/NPMC/NPMC_EmergencyMedicinePGTraining.pdf.

3. Emerg Med Australas. 2018 Dec;30(6):820-826. doi: 10.1111/1742-6723.13183. Epub 2018 Sep 25.

4. https://www.who.int/indonesia/news/detail/15-11-2022-the-republic-of-indonesia-and-who-agree-to-strengthen-health-emergency-operational-readiness-and-emergency-medical-teams-in-countries

**Appendix 3. Geographic and Demographic Differences:**

The geography and population density of Indonesia and Malaysia differ significantly, influencing their emergency medical systems, particularly Java Island, is the most densely populated area.

**Malaysia:** [1,2]

Population: Approximately 34,671,895 (2024).

Geographic Area: 330,803 square kilometers.

Population Density: 105 people per square kilometer.

**Indonesia:** [3,4]

Population: Approximately 279,930,972 (July 2024).

Geographic Area: 1,904,569 square kilometers.

Population Density: About 154 people per square kilometer.

These differences in geography and demographics must be considered when comparing the emergency medical systems of both countries.

**[References]**

1. https://www.macrotrends.net/global-metrics/countries/MYS/malaysia/population

2. <https://en.wikipedia.org/wiki/Malaysia>

3. https://www.worldometers.info/world-population/indonesia-population/

4. https://en.wikipedia.org/wiki/Geography_of_Indonesia

**Supplemental Figure 1. Participating hospitals**


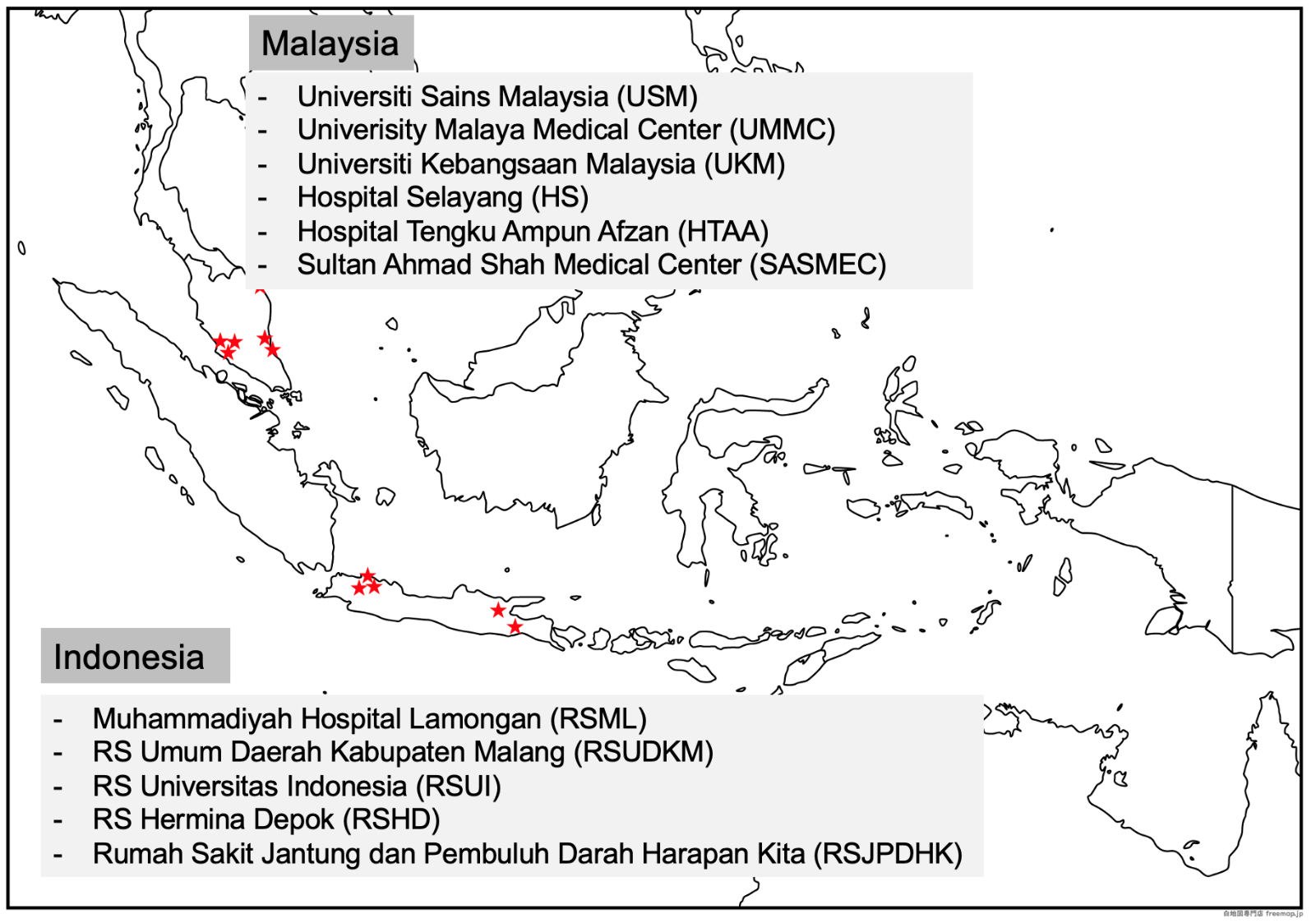


Pre-intervention phase

**Online Survey on the current status and standard of emergency medicine and trauma services at the emergency department in Indonesia**

To all who are interested in participating in the pilot project with TXP Medical Co. Ltd. and Department of Emergency Medicine in Indonesia.

The objective of this research is to investigate the current status of the Indonesian emergency medicine and trauma care system before the implementation of the NSER mobile in prehospital care. Your contribution to this study is invaluable for the improvement of the emergency department (ED).

Please read through the description of the survey below and click "Participate" at the bottom of the page to proceed to the survey.

**Preliminary Confirmation on study participation**

1. This survey consists of two main sections, "Respondents Demographic Characteristics" and "Survey on the emergency department," with a total of 20 questions (estimated time: 10 minutes).
2. There will be no patient information involved in this study only your honest opinions on the current ED. We appreciate your candid responses.
3. Participation in this survey is voluntary. This survey can be dropped at any time if you decide not to participate.
4. Survey respondents are guaranteed the right to refuse to respond and will not be disadvantaged or compelled to respond if they change their minds and wish to terminate their response midway through the survey.
5. Ethical approval (number) was obtained on “date”.

If you have any questions or concerns about this survey or its ethics, please do not hesitate to contact the principal investigator. We will sincerely try to answer and resolve your questions.

 Principal Investigator of this survey:

Keibun Liu (TXP Medical Co. Ltd): [keibun.liu@txpmedical.com](mailto:keibun.liu@txpmedical.com)

Is this the first time you answer this questionnaire? (This question aims to prevent duplicate responses from the same person.)

 □Yes □No (Choose "Yes" to continue, "No" to end)

For those who selected “Yes”, if you agree to participate after reading the above, please click **“Agree to participate”** at the bottom of this web page to start the questionnaire. If you do not agree to participate in this survey, please click **“Disagree to participate”** at the bottom to close this web page.

 □Agree to participate □Disagree to participate

| **[Respondents demographic characteristics]** |
| --- |
| Please write the first 5 characters of your email address you received this survey from. This information will be used as a unique key for future surveys. If you have less than 5 characters in your email address, please write as many as possible.  For example, if your email address is “keibun@”, the answer would be “keibu”.  If you email address is “keib@”, the answer would be “keib”. |
| 1. Age (years)   - ~19 years old - 20~29 years old - 30~39 years old - 40~49 years old - 50~59 years old - 60 years old or older |
| 2. Gender   - Male - Female |
| 3. Occupation   - Emergency physician (Certified) - Emergency physician (Non-certified) - Medical officer - Assistant Medical Officer (AMO) - Nurse - Emergency Medical Technician (EMT) - Other (free text) |
| 4. Years of experience in your current position (years) |
| 1. Current position: 2. AMO U29 or equivalent 3. Nurse U29 or equivalent 4. AMO U32 and above 5. Nurse U32 and above 6. Medical officer any grades 7. Emergency physician |
| 1. Highest academic qualification 2. Diploma 3. Undergraduate degree 4. Master’s degree 5. PhD |

| **［Survey on the emergency department (ED)]** |
| --- |
| 1. **Outpatient** care in the ED provided by your center is in accordance to national standard.  Non-critical cases are managed in ED according to national standard   \| 1 \| 2 \| 3 \| 4 \| 5 \| 6 \| 7 \| 8 \| 9 \| 10 \| \| --- \| --- \| --- \| --- \| --- \| --- \| --- \| --- \| --- \| --- \|   Strongly disagree Strongly Agree  Critical cases are managed in ED according to national standard   \| 1 \| 2 \| 3 \| 4 \| 5 \| 6 \| 7 \| 8 \| 9 \| 10 \| \| --- \| --- \| --- \| --- \| --- \| --- \| --- \| --- \| --- \| --- \|   Strongly disagree Strongly Agree |
| 2. What are the issues related to the ED system?  (PHC: pre-hospital care)   - Decision made by call center staff to accept a patient from the pre-hospital care (PHC) is Time-consuming  \| 1 \| 2 \| 3 \| 4 \| 5 \| 6 \| 7 \| 8 \| 9 \| 10 \| \| --- \| --- \| --- \| --- \| --- \| --- \| --- \| --- \| --- \| --- \|   Strongly Disagree Strongly Agree   - Inadequate clinical information from the pre-hospital care (PHC)  \| 1 \| 2 \| 3 \| 4 \| 5 \| 6 \| 7 \| 8 \| 9 \| 10 \| \| --- \| --- \| --- \| --- \| --- \| --- \| --- \| --- \| --- \| --- \|   Strongly Disagree Strongly Agree   - To record patient information manually from the pre-hospital care (PHC) is Time-consuming  \| 1 \| 2 \| 3 \| 4 \| 5 \| 6 \| 7 \| 8 \| 9 \| 10 \| \| --- \| --- \| --- \| --- \| --- \| --- \| --- \| --- \| --- \| --- \|   Strongly Disagree Strongly Agree  Preparing medications and equipment to treat medical emergencies prior to patient arrival is common (e.g., thrombolysis for stroke protocol, streptokinase for acute myocardial infarction   \| 1 \| 2 \| 3 \| 4 \| 5 \| 6 \| 7 \| 8 \| 9 \| 10 \| \| --- \| --- \| --- \| --- \| --- \| --- \| --- \| --- \| --- \| --- \|   Strongly Disagree Strongly Agree   - Inefficient system to control and manage patient flow in ED  \| 1 \| 2 \| 3 \| 4 \| 5 \| 6 \| 7 \| 8 \| 9 \| 10 \| \| --- \| --- \| --- \| --- \| --- \| --- \| --- \| --- \| --- \| --- \|   Strongly Disagree Strongly Agree   - ED overcrowding/ access block is common on a daily basis  \| 1 \| 2 \| 3 \| 4 \| 5 \| 6 \| 7 \| 8 \| 9 \| 10 \| \| --- \| --- \| --- \| --- \| --- \| --- \| --- \| --- \| --- \| --- \|   Strongly Disagree Strongly Agree   - Inefficient triage system  \| 1 \| 2 \| 3 \| 4 \| 5 \| 6 \| 7 \| 8 \| 9 \| 10 \| \| --- \| --- \| --- \| --- \| --- \| --- \| --- \| --- \| --- \| --- \|   Strongly Disagree Strongly Agree   - To enter patient information into the electronic medical record is Time-consuming  \| 1 \| 2 \| 3 \| 4 \| 5 \| 6 \| 7 \| 8 \| 9 \| 10 \| \| --- \| --- \| --- \| --- \| --- \| --- \| --- \| --- \| --- \| --- \|   Strongly Disagree Strongly Agree   - Others (free text) |
| 3. The quality of patient information received from the pre-hospital care (PHC) to the ED is adequate/enough.   \| 1 \| 2 \| 3 \| 4 \| 5 \| 6 \| 7 \| 8 \| 9 \| 10 \| \| --- \| --- \| --- \| --- \| --- \| --- \| --- \| --- \| --- \| --- \|   Strongly Disagree Strongly Agree |
| 4. What information are commonly missing in pre-hospital care (PHC) report? (Click all that apply)   - Main symptoms  \| 1 \| 2 \| 3 \| 4 \| 5 \| 6 \| 7 \| 8 \| 9 \| 10 \| \| --- \| --- \| --- \| --- \| --- \| --- \| --- \| --- \| --- \| --- \|   Strongly Disagree Strongly Agree   - Vital signs  \| 1 \| 2 \| 3 \| 4 \| 5 \| 6 \| 7 \| 8 \| 9 \| 10 \| \| --- \| --- \| --- \| --- \| --- \| --- \| --- \| --- \| --- \| --- \|   Strongly Disagree Strongly Agree   - History of presenting illness  \| 1 \| 2 \| 3 \| 4 \| 5 \| 6 \| 7 \| 8 \| 9 \| 10 \| \| --- \| --- \| --- \| --- \| --- \| --- \| --- \| --- \| --- \| --- \|   Strongly Disagree Strongly Agree   - Comorbidity  \| 1 \| 2 \| 3 \| 4 \| 5 \| 6 \| 7 \| 8 \| 9 \| 10 \| \| --- \| --- \| --- \| --- \| --- \| --- \| --- \| --- \| --- \| --- \|   Strongly Disagree Strongly Agree   - Past medical history  \| 1 \| 2 \| 3 \| 4 \| 5 \| 6 \| 7 \| 8 \| 9 \| 10 \| \| --- \| --- \| --- \| --- \| --- \| --- \| --- \| --- \| --- \| --- \|   Strongly Disagree Strongly Agree   - Medication history  \| 1 \| 2 \| 3 \| 4 \| 5 \| 6 \| 7 \| 8 \| 9 \| 10 \| \| --- \| --- \| --- \| --- \| --- \| --- \| --- \| --- \| --- \| --- \|   Strongly Disagree Strongly Agree   - Family history  \| 1 \| 2 \| 3 \| 4 \| 5 \| 6 \| 7 \| 8 \| 9 \| 10 \| \| --- \| --- \| --- \| --- \| --- \| --- \| --- \| --- \| --- \| --- \|   Strongly Disagree Strongly Agree   - Clinical Progress of patient during pre-hospital care (PHC)  \| 1 \| 2 \| 3 \| 4 \| 5 \| 6 \| 7 \| 8 \| 9 \| 10 \| \| --- \| --- \| --- \| --- \| --- \| --- \| --- \| --- \| --- \| --- \|   Strongly Disagree Strongly Agree   - Images of the patient and scene  \| 1 \| 2 \| 3 \| 4 \| 5 \| 6 \| 7 \| 8 \| 9 \| 10 \| \| --- \| --- \| --- \| --- \| --- \| --- \| --- \| --- \| --- \| --- \|   Strongly Disagree Strongly Agree   - Location of scene  \| 1 \| 2 \| 3 \| 4 \| 5 \| 6 \| 7 \| 8 \| 9 \| 10 \| \| --- \| --- \| --- \| --- \| --- \| --- \| --- \| --- \| --- \| --- \|   Strongly Disagree Strongly Agree   - Estimated time of arrival to hospital  \| 1 \| 2 \| 3 \| 4 \| 5 \| 6 \| 7 \| 8 \| 9 \| 10 \| \| --- \| --- \| --- \| --- \| --- \| --- \| --- \| --- \| --- \| --- \|   Strongly Disagree Strongly Agree  Others (free text) |
| 1. From the following options, rate the possible problems in communication from pre-hospital care (PHC) to the ED/ call center.  - Inefficient tools to transfer patient information from pre-hospital care (PHC) to ED PRIOR to arrival (i.e., during transportation)  \| 1 \| 2 \| 3 \| 4 \| 5 \| 6 \| 7 \| 8 \| 9 \| 10 \| \| --- \| --- \| --- \| --- \| --- \| --- \| --- \| --- \| --- \| --- \|   Strongly Disagree Strongly Agree   - Inefficient systems to receive and transcribe the patient information from pre-hospital care (PHC) to the hospital medical record  \| 1 \| 2 \| 3 \| 4 \| 5 \| 6 \| 7 \| 8 \| 9 \| 10 \| \| --- \| --- \| --- \| --- \| --- \| --- \| --- \| --- \| --- \| --- \|   Strongly Disagree Strongly Agree   - Inaccurate pre-hospital care (PHC) information is common (missing, incorrect information is sometimes given, information is mixed up with other patients, etc.)  \| 1 \| 2 \| 3 \| 4 \| 5 \| 6 \| 7 \| 8 \| 9 \| 10 \| \| --- \| --- \| --- \| --- \| --- \| --- \| --- \| --- \| --- \| --- \|   Strongly Disagree Strongly Agree   - Communication with pre-hospital care (PHC) team is Time-consuming  \| 1 \| 2 \| 3 \| 4 \| 5 \| 6 \| 7 \| 8 \| 9 \| 10 \| \| --- \| --- \| --- \| --- \| --- \| --- \| --- \| --- \| --- \| --- \|   Strongly Disagree Strongly Agree   - Others (free text) |
| 6. When do you prepare the medications and equipment necessary for medical emergency prior to patient arrival to the ED? (Click all that apply)   - Immediately after receiving the call from pre-hospital care (PHC) - After the patient arrives to the ED - Not prepared - Others (Free text) |
| 7. The quality of the current patient triage system in the ED is…   \| 1 \| 2 \| 3 \| 4 \| 5 \| 6 \| 7 \| 8 \| 9 \| 10 \| \| --- \| --- \| --- \| --- \| --- \| --- \| --- \| --- \| --- \| --- \|   Not working at all Excellent |
| 8. How are pre-hospital care (PHC) information handled upon patient arrival to the receiving hospital?   - The information will not be stored in the hospital database - The information will be stored in the hospital paper based database - The information will be stored in the electronic medical record - Others (free text) |
| 9. The current effort to enter patient information into the electronic medical record in the ED is…   \| 1 \| 2 \| 3 \| 4 \| 5 \| 6 \| 7 \| 8 \| 9 \| 10 \| \| --- \| --- \| --- \| --- \| --- \| --- \| --- \| --- \| --- \| --- \|   Very easy Time-consuming |
| 10. How crowded is the ED during these shift periods?  Morning shift (i.e., 0700–1400hours)   \| 1 \| 2 \| 3 \| 4 \| 5 \| 6 \| 7 \| 8 \| 9 \| 10 \| \| --- \| --- \| --- \| --- \| --- \| --- \| --- \| --- \| --- \| --- \|   Least crowded Most crowded  Afternoon shift (i.e., 1400–2100hours)   \| 1 \| 2 \| 3 \| 4 \| 5 \| 6 \| 7 \| 8 \| 9 \| 10 \| \| --- \| --- \| --- \| --- \| --- \| --- \| --- \| --- \| --- \| --- \|   Least crowded Most crowded  Night shift (i.e., 2100-0700hours)   \| 1 \| 2 \| 3 \| 4 \| 5 \| 6 \| 7 \| 8 \| 9 \| 10 \| \| --- \| --- \| --- \| --- \| --- \| --- \| --- \| --- \| --- \| --- \|   Least crowded Most crowded |
| 11. The decision to decant patient to another hospital for further investigations or treatments from the ED is…   \| 1 \| 2 \| 3 \| 4 \| 5 \| 6 \| 7 \| 8 \| 9 \| 10 \| \| --- \| --- \| --- \| --- \| --- \| --- \| --- \| --- \| --- \| --- \|   Very easy Time-consuming |
| 12. The current effort involved in writing a referral letter, when the patient needs to be transported to another hospital for further investigations or treatments from the ED is…   \| 1 \| 2 \| 3 \| 4 \| 5 \| 6 \| 7 \| 8 \| 9 \| 10 \| \| --- \| --- \| --- \| --- \| --- \| --- \| --- \| --- \| --- \| --- \|   Very easy Time-consuming |
| 13. The patient’s length of stay in ED is…   \| 1 \| 2 \| 3 \| 4 \| 5 \| 6 \| 7 \| 8 \| 9 \| 10 \| \| --- \| --- \| --- \| --- \| --- \| --- \| --- \| --- \| --- \| --- \|   Very short Very long |
| 14. From the following options, rate each factor that may contribute to the patient’s ED length of stay   - Time to record the patient’s information from the pre-hospital care (PHC) team  \| 1 \| 2 \| 3 \| 4 \| 5 \| 6 \| 7 \| 8 \| 9 \| 10 \| \| --- \| --- \| --- \| --- \| --- \| --- \| --- \| --- \| --- \| --- \|   Very short Very long   - Taking medical history  \| 1 \| 2 \| 3 \| 4 \| 5 \| 6 \| 7 \| 8 \| 9 \| 10 \| \| --- \| --- \| --- \| --- \| --- \| --- \| --- \| --- \| --- \| --- \|   Very short Very long   - Physical examination  \| 1 \| 2 \| 3 \| 4 \| 5 \| 6 \| 7 \| 8 \| 9 \| 10 \| \| --- \| --- \| --- \| --- \| --- \| --- \| --- \| --- \| --- \| --- \|   Very short Very long   - Investigations and their turn around time (e.g., laboratory test, X-ray, etc.)  \| 1 \| 2 \| 3 \| 4 \| 5 \| 6 \| 7 \| 8 \| 9 \| 10 \| \| --- \| --- \| --- \| --- \| --- \| --- \| --- \| --- \| --- \| --- \|   Very short Very long   - Entering information into the electronic medical record or manual documentation  \| 1 \| 2 \| 3 \| 4 \| 5 \| 6 \| 7 \| 8 \| 9 \| 10 \| \| --- \| --- \| --- \| --- \| --- \| --- \| --- \| --- \| --- \| --- \|   Very short Very long   - Patient and family counselling  \| 1 \| 2 \| 3 \| 4 \| 5 \| 6 \| 7 \| 8 \| 9 \| 10 \| \| --- \| --- \| --- \| --- \| --- \| --- \| --- \| --- \| --- \| --- \|   Very short Very long   - Other (free text) |
